# Supplementary material for: How does COVID-19 affect electoral participation? evidence from the French municipal elections
Source: PLoS One. 2021 Feb 24;16(2):e0247026. doi: 10.1371/journal.pone.0247026 (PMC7904179; doi:10.1371/journal.pone.0247026)
Supplement: S1 Appendix — (PDF) [file pone.0247026.s001.pdf]

How Does COVID-19 Affect Electoral Participation? Evidence from the French Municipal Elections.
S1 Appendix

Abdul Noury*, Abel François, Olivier Gergaud, Alexandre Garel

December 31, 2020

S1 Expected effects of COVID-19 on turnout

According to the calculus of voting framework, a disease should have a negative impact on electoral participation through few modifications into the rational calculus of voting. Traditionally, following Riker (1), the calculus of voting is represented by the following equation:

$$R = Bp_d - C + D \tag{1}$$

where the total reward from voting (R) depends on the benefit associated with the electoral outcome (B) times the subjective probability of being the decisive voter (p_d), plus the satisfaction of voting regardless election outcome (D) compared to the independent of the outcome costs incurred in voting (C). [1] Inclusion of epidemic disease leads to two changes. First, we have to distinguish infected people from the other, because the former have a higher cost of voting than the other even if the component of the cost are the same[2]. Namely, $C_{infected} > C_{no-infected}$ inducing $R_{infected} < R_{no-infected}$ and a lower electoral participation for infected people.

Second, for non infected people, an additional cost appears which comes from the consequence of the disease and is related to their probability of being infecting through voting operation. So, in the calculus become

$$R = Bp_d - (C + p_e C_e) + D \tag{2}$$

where p_e is the probability of being contaminated by casting a ballot in the polling station and C_e is the cost of catching the disease. The probability of being infected (p_e) has to

*Corresponding Author: Abdul Noury; E-mail: agn2@nyu.edu

[1]For a large survey of the literature dedicated to the economics of voting, see (2), (3), (4) and (5).

[2]If infected and ill voters take into account the prejudice they can produce by infected other people into the polling station, the cost is even higher.

be assessed by the voter to make his decision. In particular, it depends on the disease prevalence around the voter. The disease-related cost depends on two elements: virus characteristics and voters characteristics. According to the hazardousness of the virus, infected people bear an opportunity cost (C_e), mainly a loss of income. For instance, a cold has not the same consequence as Ebola. But the cost also depends on the person characteristics. For instance, a cold has not the same consequence for a healthy person as for a frail person.

Moreover, if people take into account the impact of their health situation on their relative, we add another cost into the calculus. This is also an expected cost because it is related to the probability of being infected by voting. And we assume the voter bears a cost deriving directly from the health deterioration of their relatives due to his voting decision. So, the disease-related cost becomes $p_e(C_e + \alpha C_r)$ where α is an indicator of altruism and C_r the cost for relatives.

Finally, the impact of epidemic disease rests on three distinct mechanisms: an ex post mechanism through the infection of voters, an ex-ante mechanism through the risk of infection for voters, and a side effect through the effective or potential infection of the voters relatives. And we can make three predictions about electoral participation in time of epidemic disease. First, we expect a lower turnout rate. Second, the magnitude of this reduction depends on voter characteristics, especially health –i.e. already infected– and age. Third, the magnitude also depends on the subjective probability of being infected by virus when casting a ballot.

S2 Survey of the extant literature on health and turnout

In general, health has received relatively little attention as an empirical factor of voter participation, and epidemic disease spread even less.[3] According to the data scrutinized, we can distinguish studies on individual-level data from aggregated-level data. The definition and measurement of health condition and situation also vary across the papers.

Studies based on individual data most of the time measures health through a self-reported evaluation made by respondents.[4] Moreover, this measurement rests on usual and general health condition and is not related to specific period or disease. For instance, (7; 8; 9; 10; 11; 12) show poor self-rated health is correlated with less individual electoral participation for various elections and contexts. The mechanism behind the relationship is that poor health is related to a weaker social network that leads to less social participation, in particular political and electoral participation. With more sophistication as the relationship seems to be conditioned to several respondent's characteristics, this link is also observed for oldest people (13) and youngest people (14). In a very close perspective, disabled people also participate substantially less than the average population in community affairs, including voting, as suggested by the works of Schur and her co-

[3] We exclude from our survey the papers dealing with the impacts of natural disaster on electoral behavior, such as Katrina for instance.

[4] For a synthetic presentation of outcomes from literature using individual data, see (6).

autors (15; 16; 17; 18). This population experiences a higher cost of voting because of their difficulties to move and the lack of adaptation of polling station and voting process to their disabilities; and this higher cost is invariant in time. When studies success to refine respondents' health situation, the relationship appears as more subtle according to the health problems. For instance, chronicle patients with alcoholism and mental disorders[5] participate less at election, and patients with cancer and/or asthma, for instance, vote more (20) and those with heart disease vote less (21). As a result, combined and multiple health conditions have a detrimental effect on electoral participation (20), and long-term multiple illness have negative impact that is stronger than health problem occurred in the year before the election (22).

Recently an emerging literature focuses on the consequences of the COVID-19 crisis on politics. Based on survey data, they generally find a positive impact of the crisis. (23) examine the effect of health crisis on political behavior in Bavaria, and report that the dominant party in Bavaria benefited from the COVID-19 crisis. Similarly, (24) use a series of survey experiments and social media data in Canada to show that the crisis is positively correlated with greater support for the government. (25) report, however, that in Spain the COVID-19 crisis is associated with a national bias and higher demand for a strong technocratic and authoritarian policy-making.

Using survey data from Western European countries (26) compare political attitudes of respondents before and after a national lock-down. They find that the lock-down increased support for the status quo decision-makers, institutions and regimes. But they don't find any effect of the lock-down on ideology or political interest. Closely related, and sharply in contrast, to our analysis is their question about turnout. They report that turnout would increase from 81% to 83% after the lock-down. It remains to be seen if people maintain their response as social and economic costs of the pandemic rise, particularly in a context where governments adopt very different approaches to combat the pandemic.

Now, we turn to aggregated data that seem more suitable to analyze impacts of epidemic disease, and so, more in line with our purpose. Indeed, it is unlikely that a survey includes enough people being infected by the disease to allow testing its impact on electoral participation. A unique study scrutinizes such relationship at aggregated level and has been written by (27). He examines at regional level the link between turnout rate and local influenza prevalence in Finland and the United States from 1995 to 2015. And he concludes that influenza outbreaks are associated with lower electoral participation in both countries. In his study, the author is not able to distinguish between the three effects of epidemic disease on participation detailed before. Moreover, as he measures the influenza prevalence at the election moment, his measure is mainly about an ex post measurement.

All those studies analyze either the specific decision of patient in normal time, either the impact of decease on electoral turnout through the direct infection of voters and the higher cost of voting for infected voters. Alternatively, they do not take into account, theoretically or empirically, that voting itself is a source of infection which modifies calculus of voting

[5] Several studies show a negative relationship between electoral participation and mental health, especially depression (e.g. 19)

and participation decision of healthy people. Given the COVID-19 situation at time of 2020 French election, we propose to mainly study the impact of likely cost of voting related to the epidemic decease.

S3 COVID-19 outbreak in France

In this section, we detail the facts as well as French government's policy responses on the COVID-19 outbreak in France. The purpose is to know at what level of the outbreak development and spread the 2020 municipal election hold.

S3.1 Chronicle of COVID-19 spread in France

The chronicle of COVID-19 in France and the time location of the 2020 municipal elections can be summarized by Figure S1. We see that the election takes place at the very beginning of the COVID-19 outbreak.

Figure S1: **COVID-19 cases in France.**

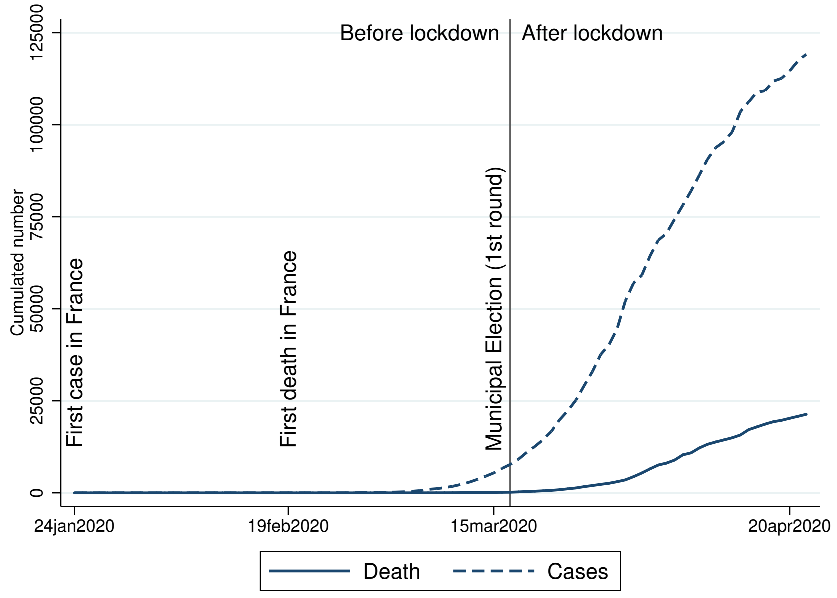

The first French -and European- case was confirmed in south-west on January 24th.[6] A young couple, an old man and his sister were also confirmed at this period, but they all were back from China. The old man deceases at mid-February and were the first death from covid-19 outside Asia. It seems that those first cases were efficiently contained. From there, several unrelated clusters appeared during February month.

[6]Finally, we know now that the very first French patients have been hospitalized at mid-November 2019. But at this date they have not been detected as COVID-19 infected.

On Thursday, March 12th, 2020, French President Emmanuel Macron announced at the end of his first TV appearance in the wake of the COVID-19 sanitary crisis, his decision to maintain the first round of the municipal elections initially scheduled on Sunday, March 15. Over the same interview, Emmanuel Macron declared France ‘at War’ with virus. At that time, the number of cases was doubling every three days in the country and the saturation of the French hospital system was a credible threat. The elderly, a population facing a higher risk of dying of the virus, was invited to stay home. Two days later, French Prime Minister Édouard Philippe announced in a televised press conference that starting at midnight on Saturday, March 14th all businesses that are not deemed essential, including restaurants, cafes, cinemas and clubs, would be closed to increase social distancing. While Emmanuel Macron was not supporting the idea of holding the poll as initially scheduled, he had to deal with most opposition parties and even some of his party members (La République en Marche) who strongly oppose postponing it. One day after the poll, President Macron announced a strict 15-day lock-down for France and his decision to postpone the second round of the election to a more favorable period, probably in June or later in the Fall.

From a more detailed perspective, we observe a strong acceleration of the public decision just before the election. Until the 1st round of the municipal election holing Sunday March 15th, national government increasingly reactions to the outbreak were

- 5th March, ban on gatherings of more than 5,000 people in an enclosed space;
- 10th March, ban on gatherings of more than 1,000 people, whatever the characteristics of the space;
- 14th March, ban on gatherings of more than 100 people, whatever the characteristics of the space;
- 15th March, closure of the most part of public establishments; only essential services and public utilities remain open;
- 16th March, closure of schools and higher education establishments, ban on religious gatherings;
- 17th March (at noon), beginning of the lock-down with strong travel restriction.

S3.2 Spread of COVID-19 in France

The maps in Figure S2 illustrate the spread of COVID-19 using three distinct measures: number of declared COVID-19 cases, number of hospitalizations due to coronavirus, and number of emergency visits at a hospital. In addition, those maps indicate the location of COVID-19 clusters as of March 15.

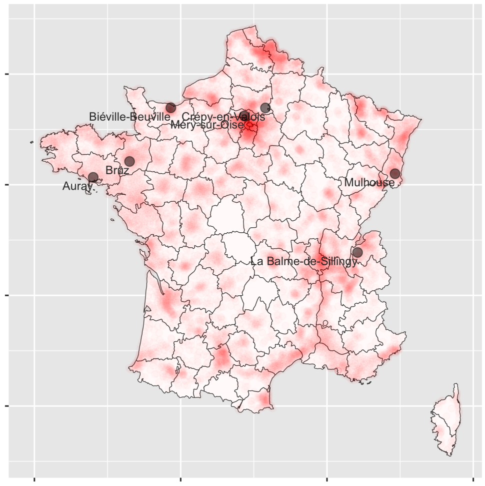

(a) Number of Cases

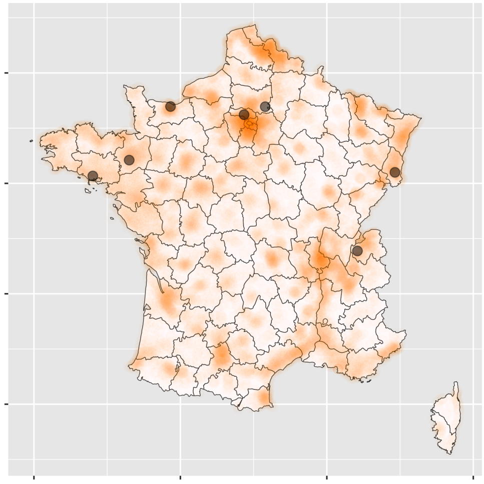

(b) Number of Hospitalizations

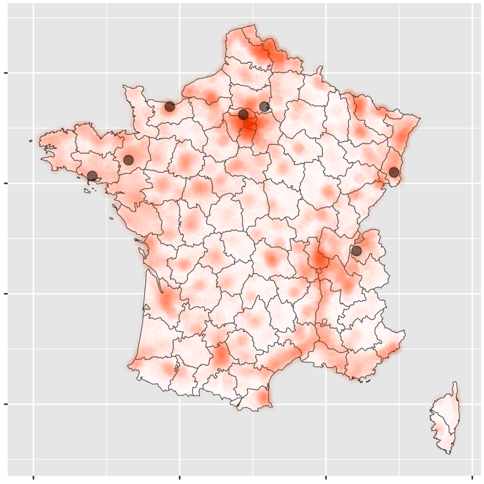

(c) Number of Emergencies

Figure S2: Spatial distribution of COVID-19 and first Clusters

S4 The 2020 French municipal election

In this section, we detail the role of the French municipal governments and how it is elected. We also give some elements of context for the 2020 election.

S4.1 French municipal election and government

Municipal government is the lowest and smallest layer of government in France.[7] It consists in the municipal council which appoints the executive branch: the mayor and his deputies. The municipal council is elected by registered voters of the city every 6 years and according to a two-round voting system which the definition changes according to the size of the city, defined by the number of inhabitants. If the city has less than 1,000 inhabitants, a plurality-at-large voting system in two rounds with panachage is used. The candidate does not have to be registered to be elected. If the city has more than 1,000 inhabitants, the council members are elected using a plurinominal proportional representation with two rounds. The winning list obtaining the majority of votes at the either first or second round -if necessary- has the majority of seats at the municipal council.

Beyond it is important to note that there is no vote by mailing or Internet in France. In other words, it is not possible to avoid voting travel to the polling station. Moreover, absentee voting is very complicated and limited. For instance, to establish the absentee the voter has to file the administrative document at a police station, several days before the election. As a result, absentee voting is hardly used in French elections and do not avoid social interaction and risk of contamination. For the 2020 elections, it was very difficult to anticipate the spread of the virus given its fast spread and the sudden reactions of government.

Because French political system is highly centralized, municipal council is in charge with very local public policies. Mainly it manages urban, land and real estate policies and public transport as well. At the date of the election, there were about 36,000 municipalities in France.

S4.2 Political context of the election

The 2020 municipal elections hold almost three years after the 2017 presidential election and one year before other 2021 local elections. If we push away the 2019 European elections that are second order elections, the municipal elections can be considered as the first mid-term elections of the incumbent President. They can be seen by voters as a way of punishing or rewarding national government, even if this link is not clear into the academic literature.

Moreover, all the actors, especially local candidates and national government, try to wipe out the national dimension of the election. For instance, candidates are using to suppress all partisan signals -i.e. national signals- in their propaganda materials. As a

[7]There are three other local governments: *inter-municipal* that is a cooperation of municipalities, *Conseils Départementaux* that manage public policies at departement level and *Conseil Régionaux* at region level. Other government bodies are national.

result, the campaign is dominated by very local issues and incumbent mayors have a huge electoral advantage.

S4.3 Expected effects of COVID-19 on participation at 2020 French elections

To sum up, the COVID-19 situation in France at the election dawn was as follows.

1. There were only few cases who were detected and known, but it seemed the virus already spread among population;
2. The cases came mainly from several places, meaning the outbreak was limited in France to a small number of clusters;
3. Medical information published suggested oldest people face more COVID-19-related health risks;
4. Municipal elections toke place at the very beginning of the outbreak;

Those facts have heavy conclusions for our empirical work and following the distinction made in our theoretical background. First, we can ignore the impact of already infected people on electoral turnout, because they are only few at election time. Second, we have to focus on cost and probability of being infected for voters. Third, the available information for people to assess their risk of infection and the related cost are very sparse.

We apply the theoretical impacts of epidemic disease detailed previously to the 2020 french electoral situation. First, we do not assume an ex post mechanism through people already infected by the COVID-19, because from an empirical point f view it has to be tiny. And consequently, main of the COVID-19 impact on electoral participation rests on an ex-ante mechanism through the risk of infection by voting. Second, probability of being infected depends on the geographical spread of the virus which was very concentrated at the election time. And the cost of being infected depends on voters' age.

So, in terms of prediction, we expect a lower turnout rate because of the general increase of cost of voting. We also expect the reduction of participation is related to voters' age and to probability of being infected by virus when casting a ballot.

S5 Dataset

In this section we first describe the observations -i.e. the municipalities- included in our studied sample. Then we present the variables related to COVID-19 and measuring the likely cost of being, on the one hand, and the variables corresponding to the other elements of calculus of voting, on the other hand.

S5.1 Data sources

Municipal election data for 2014 and 2020 (e.g., ballots, registered voters, turnout) are collected from the French Ministry of Internal Affairs[8]. For each municipality, we retrieve sociodemographic and geographic data at the municipality and department levels from INSEE, the French National Institute of Statistics and Economic Studies [9]. The number of cases and deaths associated with COVID-19 at the level of each department as of the 14/03/2020 are gathered from the regional public health agencies[10]. Data at the department level on the number of COVID-19 related medical acts, emergency visits, and hospitalizations are from France's public health agency [11].

S5.2 Municipalities in our sample and turnout rate

To compare electoral turnout across municipalities, we exclude from our studied sample municipalities with less than 1,000 inhabitants. Put differently, all the studied municipalities have the same voting system in our observations sample.

Moreover, we decided to exclude municipalities from oversea territories and Corsica. Corsica is a French island close to Italy with specific habits in particular in terms of registration. Lot of Corsica-originated people live in the French part of the continent but are still registered as voters in Corsica. It implies long trip and heavy management of absentee. Oversea territories have also cultural specificities and patterns, moreover for them lot of socio-demographic variables are not available.

Lastly, we also decide to exclude the three largest French municipalities: Paris, Lyon and Marseille. The reason is also related to institutional characteristics: these municipalities are split in arrondissements or *secteurs* that are small municipalities with very restricted responsibilities. There are a council and a mayor for each arrondissement or *secteur*, and a part of the councillors seat in the municipal council which is the overall council and elect the municipality mayor. In other words, the mayor of Paris, or Lyon or Marseille are indirectly elected by an electoral college consisting of a part of arrondissement or *secteur* councillors. So, Paris, Lyon or Marseille voters have different strategy of votes when they make their decision compared to any other French municipality, because they think about the elections simultaneously at both arrondissement or *secteur* level and municipal level.

As a result, and depending on data availability, our final sample contains around 9,300 municipalities out of the 36,500 existing municipalities. To measure the turnout, we use usual turnout rate defined as the number of voters -including the blank and null votes- divided by the number of registered voters. In our municipalities, we observe a lower

[8]Source : https://www.data.gouv.fr/fr/datasets/elections-municipales-2020-resultats/ and https://www.data.gouv.fr/fr/datasets/elections-municipales-2014-resultats-1er-tour/

[9]Source : https://www.insee.fr/

[10]Source : https://www.data.gouv.fr/fr/datasets/donnees-des-urgences-hospitalieres-et-de-sos-medecins-relatives-a-lepidemie-de-covid-19/ and https://www.data.gouv.fr/fr/datasets/donnees-hospitalieres-relatives-a-lepidemie-de-covid-19/

[11]We match election data with INSEE data using the code insee for municipality-level data and codedepartment for department-level data.

Table S1: **Turnout rate at various French municipal elections.**

	First round of elections		
Turnout rate	2020	2014	2008
$\bar{x}$	46.34	66.68	70.51
sd	11.32	8.79	8.32
Min	16.19	32.46	36.05
Max	88.52	98.22	100

$$\textit{Turnout rate} = \frac{\textit{ballots}}{\textit{registered voters}} \times 100$$

9,305 French municipalities with more than 1000 inhab.,
excluding French overseas territories, Corsica and Paris, Lyon and Marseille

turnout rate at 2020 municipal election, compared to the two previous ones (Table S1) . In average, the rate diminishes of more than twenty points of percentage between 2020 and 2014. The change between 2008 and 2014 is smaller, less than 4 points. So, the trend of turnout rate is declining but it deeply accelerates since the 2014.

To summarize, we observe a huge decline of electoral participation at the 2020 election and an increase of its variance across municipalities. If we are not able to entirely rely this huge decline to a COVID-19 effect, nevertheless we note it is in line with our general expectation about the impact of voting cost increase.

S5.3 Variables dealing with COVID-19

As the election took place at the very beginning of the epidemic outbreak, we assume that the number of infected voters was tiny. In other words, in our data, the number of affected voters is too small to impact the aggregated outcomes. We consider it as having no effect on our variables. Therefore, we have to take into account the ex ante mechanism of outbreak on voting. Variables specifically related to COVID-19 influence on electoral participation are the cost of being infected by the disease (C_e) and the probability of being infected by going to the voting station (p_e).

The information available on the COVID-19 at the very beginning of the outbreak was confused. All the characteristics of the virus and its spread were deeply discussed and debated. However, two elements emerge among published information before the election date. First, the dangerousness of the virus is strongly correlated to the age of the patient. Second, the dangerousness of the virus is also correlated to other pathology such as heart disease. So it seems that the associated pathologies are related to age, and we assume that age is the most dominant factors of COVID-19 dangerousness for people who have not specific medical knowledge. In economic terms, it means the virus cost evolves with patient age. So the cost of being infected by going to the voting station increases with patient age. At aggregated level, we measure this cost through the population structure

by age.

How to approximate the subjective likelihood of being infected by voting perceived by potential voters (i.e. p_e)? As explained below, information about COVID-19 spread was very scarce. The main public information was about the existing cluster at early March. There were, at the time of the election, 7 clusters in metropolitan France as follows: Auray, Biéville-Beuville, Bruz, Crépy-en-Valois, La Balme de Sillingy, Mulhouse and Méry-sur-Oise. The cities were located in 7 departements (code), respectively Morbihan (56), Calvados (14), Ille-et-Vilaine (35), Oise (60), Haute-Savoie (74), Haut-Rhin (68) and Val d'Oise (95). Their precise locations are presented in maps of Figure S2 That's why, we decide to proxy the probability by the geographical distance between each studied city and the nearest clusters.

Table S2: **Distance between studied cities and COVID-19 clusters.**

Nearest cluster		Distance (km)		
Municipality	Departement	Mean	s.d.	N
Auray	Morbihan	222.91	185.64	813
Biéville-Beuville	Calvados	69.70	39.38	425
Bruz	Ille-et-Vilaine	187.93	122.94	1,608
Crépy-en-Valois	Oise	117.76	50.71	1,211
La Balme de Sillingy	Haute-Savoie	230.93	137.43	3,067
Mulhouse	Haut-Rhin	104.41	56.40	882
Méry-sur-Oise	Val d'Oise	87.98	68.16	1,333
Overall		168.46	129.86	9,339

To test the sensitivty of our measure, we also use other variables that were published latter the election and that are alternative proxy of subjective probability of being infected by voting. Variable definitions, whether they are measured at municipality or department level, as well their sources are reported in Table S12.

S5.4 Variables dealing with other elements of the calculus of voting

To measure the elements of the calculus of voting that are not related to the COVID-19 outbreak, we use variables usually associated to the elements in studies on aggregated data (see ()).

To measure the benefit related to the election outcome (B) and as we work on aggregated data, we select two variables. They are closely related to the main public policy implemented by French Mayor which is land usage and housing policies. In other words, given the public policy municipal government is in charge with, we detect two population whose economic interest depends on municipal decisions: farmers who use and owns land,

on the one side, and housing owners whose real estate wealth depends on mayor decision, on the other side. So, our specification contains the proportion of farmers among municipal population and the proportion of house owners.

To measure the probability of being the decisive voter p_d, we have two variables. We consider this probability as exogenous meaning there is no strategic interaction inside electorates. We do it for at least tow reasons: first, it is pretty complicated to gauge strategic interaction with aggregated variables. Second, this assumption obtain better empirical results than strategic one (28). First, following the Downsian Closeness Hypothesis (e.g., (29)), we include into our specification, the size of electorate with the log natural transformation of registered voters in the city. Second, we take into account the number of lists running at the election as measure of electoral competition (30). This measure of election competition is better than those based on votes' distribution because of it is exogeneous and because French municipal elections are characterized by huge difference across municipalities (see Table S3). With a unique list running at the election, the election is not competitive and the expected turnout is low. With two lists, the closeness of the election is the highest because there is a unique ballot. And with three lists and more, the closeness is the lowest because with more candidates the probability of winning the election at the first round is lower and because the true choice will be made at the second round.

Moreover the competition is also more open when the incumbent mayor does not run at re-election. The variable indicating if the incumbent candidates is also a proxy of the expected closeness of the election.

Table S3: **Lists running at 2020 and 2014 French municipal elections and turnout (First round)**

	Elections		$\Delta_{2020-2014}$
	2014	2020	
Nb of lists	Municipalities (%)<br>*Average Turnout*	Municipalities (%)<br>*Average Turnout*	Changes<br>*Changes*
Unique list	2,842 (30.5)<br>*60.44*	3,511 (37,7)<br>*37.70*	+669<br>*-22.74*
2 lists	3,968 (42.6)<br>*71.61*	3,685 (39.6)<br>*54.09*	-283<br>*-17.52*
3 lists and more	2,495 (26.8)<br>*65.96*	2,109 (22.7)<br>*47.16*	-386<br>*-18.80*
Overall	9,305 (100)<br>*66.68*	9,305 (100)<br>*46.34*	-<br>*-20.34*

$$Turnout\ rate = \frac{ballots}{registered\ voters} \times 100$$

French municipalities with more than 1000 inhab.,
excluding French overseas territories, Corsica and Paris, Lyon and Marseille

To measure the benefit of voting which is not related to the election outcome (D),

we introduce several variables according to the various dimension of this elements. First, considering the sense of civic duty and satisfaction of voting *per se*, we assume that all the sociodemographics variables approximate the factors of these elements, especially education level and size of the city (inhabitants). Expressive dimension of voting is also captured by the number of list running at the election. More competing lists means more choice for voters, so they can more easily express their political preferences and they are also more motivated to vote.

Finally, to measure the standard cost of voting (C), we use several variables. First, we introduce the city density, defined as the population divided by the area of the city. In France, the regulation establishes that a voting station must not contain more than 1000 registered voters. This constraint means that the number of voting station increases linearly with the population and that more dense city have more voting station in a smaller territory inducing a smaller average distance to vote. Second, to take into account the opportunity cost of voting, i.e. time devoted both to vote decision-making and to cast a ballot, we include into the specification the median income in the city and the unemployment rate of the economic area the city belongs to. The list of variables, and their sources, and whether they are measured at municipality or department level, are reported in Table S13.

S6 Estimation Framework

In the main text we adopted a difference-in-differences approach. In order to remove the municipality fixed effects, in a second specification we estimate a model in first difference:

$$\Delta turnout_{ij} = \alpha + \beta covid_{ij} + \gamma \Delta income_{ij} + \psi \Delta list_{ij} + \Delta X'_{ij}\eta + d_j + \Delta\epsilon_{ij} \tag{3}$$

where Δx indicates the first difference (change between 2014 and 2020) of a given variable x. A major advantage of this specification is that we discard any unobserved municipality-specific heterogeneity that might drive the results of our cross-sectional analysis presented above. Here we focus on the change in turnout rate over time and aim at estimating the impact of various drivers of those changes in turnout. Our key variable of interest in this specification is again a measure related to presence and intensity of coronavirus. All other variables are included in first difference, with the set of department dummies being another exception. In this framework, we hypothesize that (a) presence of COVID-19 cases decreases turnout and (b) the decline in turnout rates is more pronounced in relatively older municipalities.

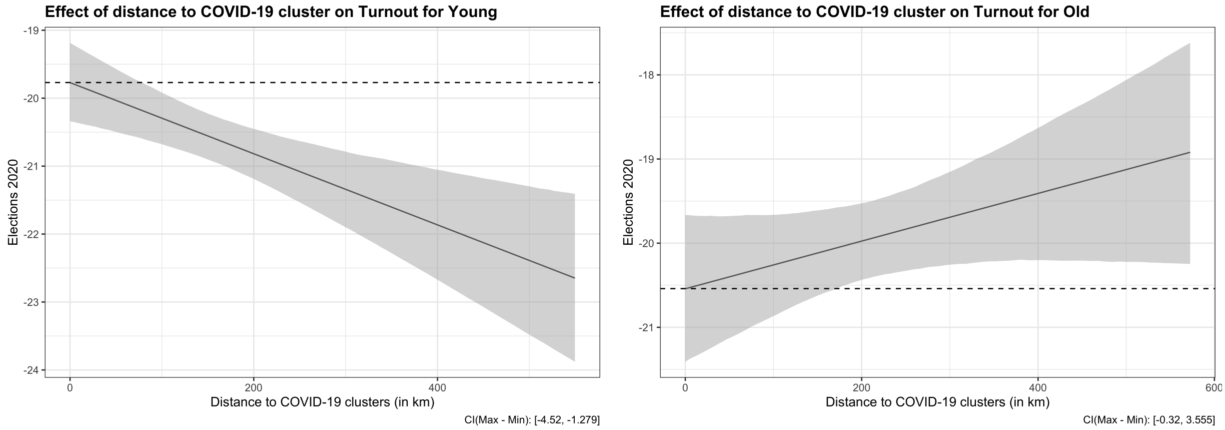

(a) Younger Municipalities

(b) Older Municipalities

Figure S3: **Effect of Distance by Sub-samples According to Age.**

S7 Estimation Results

The results of OLS regressions are reported in S4.[12] The first column shows that for the entire sample turnout sharply drops in 2020 and increases with the distance to COVID-19 clusters. The interaction between distance to clusters and post is not significant, meaning that the distance to clusters does not affect turnout differently in 2020 compared to 2014. This insignificance, however, disappears once we split the sample into two subsamples of 'young' and 'old' municipalities. A municipality is considered to be 'young' if the share of population over 65 is less than the national average, 20%, and is considered to be 'old' when that share is more than 20%.[13] The second column of Table S4 shows that the interaction effect between distance and post is negative. In contrast, column 3-7 shows that the interaction is positive and statistically significant. Thus the municipalities with higher proportions of older voters participate more (less) when they are far from (close to) COVID-19 clusters than those populated with relatively younger voters. The interaction effect becomes more significant when we include control variables, including department and municipality fixed effects. To substantively interpret the interaction effects, Figure S3 shows the turnout rate for any given level of distance to COVID-19 clusters. Turnout drops by about 20% in a municipality located closest to COVID-19 clusters, but that drop decreases to less than 18% at a municipality located on the other extreme, at the farthest distance from the disease clusters. That is, the effect of distance when moving from one extreme to another is about 2 percentage point. In contrast, for younger municipalities the decline is reinforced to about 23%.

[12] Following Conley (1999), we allow for spatial dependence of an unknown form. Although the standard errors are slightly larger than the one reported in the Table S4, the significance of coefficients did not change in any model.

[13] These results do not depend on a specific definition of old and young municipalities. We obtain qualitatively similar results when we use alternative variables such as the share population over 75.

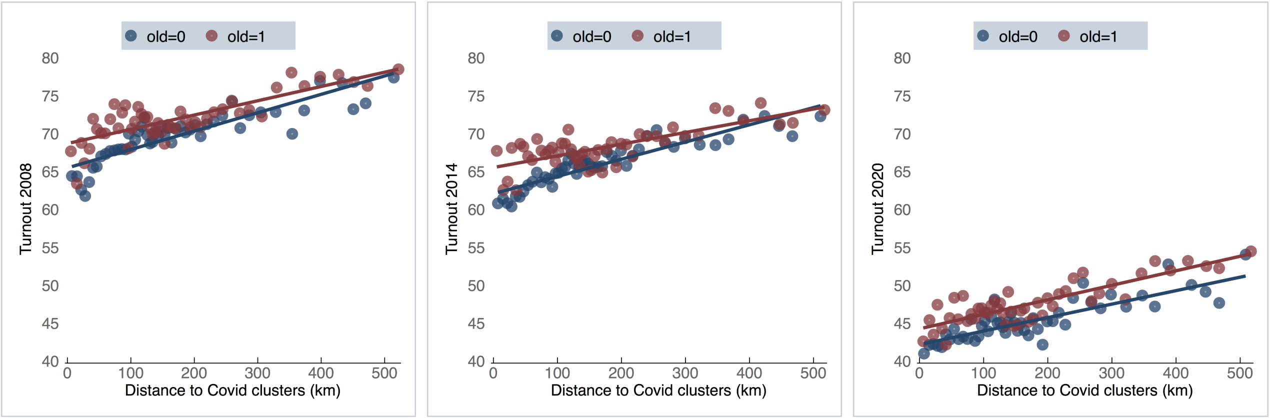

(a) 2008 Elections (b) 2014 Elections (c) 2020 Elections

Figure S4: **Binned scatter plots of distance and participation by age category.**

The approach used so far in this paper require a key identifying assumption: in the absence of COVID-19 pandemic the the relationship between turnout and distance would have remained unchanged. To assess the plausibility of this assumption we first control for a number of important variables, and second examine that relationship in previous elections, for example the 2008 local elections. Regarding controlling for important variables, columns 4-7 of Table S4 shows that the effect of distance becomes even more significant after including the set of control variables. Regarding the relationship between distance and participation, if we do not find any difference between the 2008 and 2014 elections, then it is reasonable to assume that the common trends assumption is plausible. Figure S4 shows that while both the levels and slopes are fairly similar between 2008 and 2014. The drastic difference in both level of turnout and slopes of distances is observed only in 2020. In 2008 and 2104 elections the slop for old is statistically significantly smaller than the slop for young. In 2020 elections, however, that difference in slopes is statistically insignificant (Figure S5).

The results of our first difference specification is reported in Tables S5 and S6. In Table S5 the variables measuring COVID-19 are *virus dummy* reported in columns 1-4, and *number of cases*, reported in columns 5-8. As we have already observed in Table S4, regardless of how we measure the disease prevalence the results for old and young municipalities are very different. Electoral participation decreases particularly in older towns. In contrast, in younger municipalities, it is either insignificant (column 3), or it is positive (column 7). The change in median income increases the change in turnout. Interestingly, the effect of change in electoral competition is large and significant with theoretically expected signs. Relative to a situation without any change, an increase by one or two more competing lists increases turnout by a large magnitude. Conversely, when competition decreases turnout sharply declines. The results pertaining on competitiveness of elections indicate that even during a pandemic voting calculus explains whether people vote or abstain. The results in Table S6, reflects those reported in Table S5. The only

difference is how we measure COVID-19. Here instead of presence or absence of the cases, we examine the distance from the COVID-19 clusters. Overall, the results in Table S6 support those reported in Table S5.

After comparing the 2020 elections to previous elections and having established the impact of COVID-19 on turnout, we use OLS regressions to show the determinants of turnout in 2020 elections. In Table S7 we include and then exclude a set of municipality-specific variables: size of the municipalities measured based on population (columns 1, 4-6), status of municipalities capturing whether a municipality is rural, city-center, suburb, or a remote city (columns 2,4), and municipality categories (columns 3-4). In column 5 we include the log of registered voters, and column 6 include department fixed effects. Our key variables of interests are not altered by including or excluding those control variables. Log of population density decreases turnout by a magnitude ranging from 4 to 8 percentage points. Lack of competition, measured by *liste_unique*, decreases turnout by a much larger magnitude of about 19 percentage points. Interestingly, and contrary to what one would expect, percentage of population over 75 increases turnout, a peculiar aspect of French municipal elections. As expected, home ownership, which measures the benefits of participation increases turnout. Municipalities with higher share of homeowners participate more. Political variables such as voting for Macron ($Macron\ Vote$) in the last presidential elections, and whether the incumbent mayor is a candidate, increases turnout. The economic variables such as $Median\ Income$, and $Unemployment$, are also significant, with income coefficient being negative while unemployment affecting turnout positively expect when we include department fixed effects. Given that this pandemic began in China, and as such is associated with globalization we include number of hotel rooms *per capita*, in log scale, a variable that we expect to captures presence of interest in globalization. The results reported in Table S7 shows that Number of hotel rooms *per capita* increases turnout. Obviously, in addition to turnout a key question to address is how voters voted. Did they vote for pro-globalization or anti-globalization candidates. We will analyze this important question after the second round of the elections, as it would be difficult to draw conclusions based on the first round of elections given that candidates behave strategically in the first round. A second and more important reason why we postpone this question is that at this stage the ideological positions of the candidates are not publicly available.

Table S4: **Effects of distance to COVID-19 cluster on turnout rate.**

VARIABLES	(1) All	(2) Young	(3) Old	(4) Old	(5) Old	(6) Old
post	-20.220***	-20.210***	-21.376***	-19.255***	-19.014***	-19.023***
	(0.231)	(0.283)	(0.409)	(0.268)	(0.265)	(0.247)
distance to clusters (km)	0.021***	0.021***	0.015***	0.008***	0.009***	-0.006**
	(0.001)	(0.001)	(0.001)	(0.001)	(0.001)	(0.003)
post x distance to clusters (km)	0.0002	-0.003**	0.004***	0.005***	0.004***	0.004***
	(0.001)	(0.002)	(0.002)	(0.001)	(0.001)	(0.001)
Constant	63.014***	62.471***	65.728***	110.720***	125.048***	122.341***
	(0.144)	(0.169)	(0.275)	(2.586)	(2.703)	(2.710)
Control				✓	✓	✓
Region Fixed Effects					✓	✓
Department Fixed Effects						✓
Observations	18,678	11,420	7,076	7,074	6,521	7,074
R^2	0.543	0.558	0.536	0.842	0.858	0.869

Robust standard errors in parentheses ; *** p<0.01, ** p<0.05, * p<0.1

A municipality is considered to be ‘Old’ (‘Young’) if the share population over 65 is more (less) than the national average, 20%.

S8 Robustness Checks

To analyze how stable and how robust our reported results are we perform a number of robustness checks. We first show that our results do not depend on how operationalize old and young municipalities. In Table S10 we interact *post* with *pop*65 and divide our samples within a given distance from COVID-19 clusters. Column 1 of Table S10 limits the sample to municipalities within 50 km of COVID-19 clusters, and Columns 2, 3, and 4 restrict it to those falling within 50-100 km, 100-200 km, and beyond 200 km, respectively. Columns 5-8 repeat this exercise but include a set of control variables. The results reported in that Table indicate that the coefficient of interaction between *post* and *pop*65 is negative when we consider the municipalities that are close to COVID-19 clusters. That coefficient is insignificant for intermediate municipalities that are within 50-100 km, but beyond that limit the coefficient of the interaction term becomes positive and highly significant. Overall the Table indicates that the closer a municipality is to COVID-19 cluster, relative to 2014, the lower is turnout rate for old municipalities. Conversely, the older municipalities that are far away from the COVID-19 clusters the turnout is higher than in 2014. The results become even more significant when we include control variables.

Next, we show that our results also hold when we use alternative measures of COVID-19. In Table S8, in addition to distance, we include the following measures: *Virus Dummy* a dummy indicating whether any cases of COVID-19 was observed at the time of elections, *Hospital Visits* a measure of number of hospital visits, *Hospitals Visits for corona virus* measuring the number of hospital visits due to corona virus, *Hospitalization for corona virus*, and *Corona Cases* which counts the number of COVID-19 cases in a given municipality at the time of election. Table S8 shows that, with the exception of *hospital visits*, all other variables measring COVID-19 are significant with the expected sign, positive for the distance, and negative for the presence of cases or hospital visits due to corona virus.

In Table S9 instead of a simple linear model we specify and estimate an auto-regressive model. Including the lagged dependent variable will captures factors that are important but are not observed by the analysts. The results are similar to those reported in Table S7.

Table S11 shows that when using Conley standard errors our main results are not altered. Finally, when we estimate our models using the fractional response model of (31) we obtain similar results -to be included.

Figure S5: **Determinants of Turnout in 2014 and 2020.**

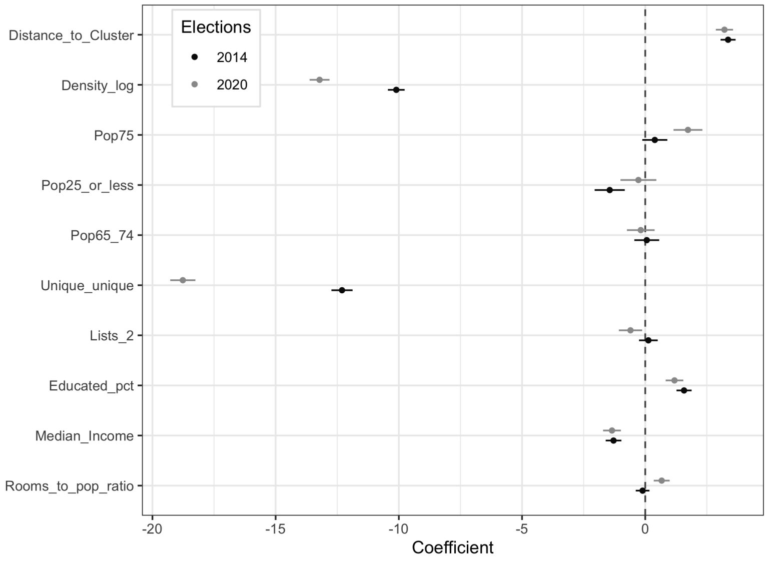

Table S5: **Determinants of ΔTurnout as a function of alternative Covid-19 incidence measures**

	(1)	(2)	(3)	(4)	(5)	(6)	(7)	(8)
VARIABLES	All	All	Young	Old	All	All	Young	Old
Virus incidence in depart. (binary)	-2.668***	-2.190***	-1.503*	-2.746***				
	(0.540)	(0.648)	(0.851)	(0.618)				
COVID-19 incidence cases nb.					-0.114*	-0.446***	0.362***	-0.679***
					(0.067)	(0.110)	(0.088)	(0.102)
Δ Population over 75 (%)	-0.407***	-0.280***	-0.477***	-0.448***	-0.402***	-0.274***	-0.494***	-0.460***
	(0.064)	(0.067)	(0.092)	(0.092)	(0.064)	(0.067)	(0.093)	(0.092)
Δ Population 25 or younger (%)	-0.207***	-0.230***	-0.186***	-0.283***	-0.204***	-0.226***	-0.165***	-0.317***
	(0.052)	(0.054)	(0.064)	(0.087)	(0.052)	(0.054)	(0.064)	(0.086)
Δ Population 65_74 (%)	-0.012	0.047	-0.134**	0.115	-0.018	0.045	-0.083	0.073
	(0.048)	(0.051)	(0.062)	(0.074)	(0.049)	(0.051)	(0.064)	(0.074)
Δ Registered Voters (log)	-8.087***	-7.392***	-7.476***	-8.523***	-8.226***	-7.407***	-7.292***	-8.812***
	(0.557)	(0.569)	(0.746)	(0.741)	(0.561)	(0.569)	(0.741)	(0.740)
Δ Median Income	0.139*	0.564***	-0.093	0.409***	0.125	0.556***	-0.012	0.311**
	(0.076)	(0.088)	(0.095)	(0.138)	(0.078)	(0.088)	(0.097)	(0.141)
Change in number of lists running (unchanged as reference):								
From 1 to 2	15.946***	15.936***	16.460***	15.137***	15.949***	15.932***	16.461***	15.117***
	(0.214)	(0.223)	(0.265)	(0.355)	(0.213)	(0.223)	(0.264)	(0.357)
From 1 to 3 and more	17.067***	17.176***	17.593***	16.459***	17.035***	17.145***	17.610***	16.369***
	(0.503)	(0.541)	(0.674)	(0.745)	(0.505)	(0.539)	(0.664)	(0.757)
From 2 to 1	-15.000***	-14.822***	-15.201***	-14.658***	-15.003***	-14.830***	-15.145***	-14.666***
	(0.160)	(0.164)	(0.206)	(0.253)	(0.160)	(0.164)	(0.208)	(0.249)
From 2 to 3 and more	3.266***	3.169***	3.475***	2.954***	3.281***	3.175***	3.475***	2.911***
	(0.206)	(0.209)	(0.288)	(0.298)	(0.207)	(0.209)	(0.287)	(0.298)
From 3 and more to 1	-15.838***	-15.749***	-16.691***	-14.782***	-15.849***	-15.756***	-16.651***	-14.831***
	(0.335)	(0.348)	(0.458)	(0.469)	(0.334)	(0.347)	(0.462)	(0.457)
From 3 and more to 2	1.077***	1.084***	0.925***	1.145***	1.071***	1.093***	0.944***	1.120***
	(0.188)	(0.192)	(0.250)	(0.283)	(0.189)	(0.192)	(0.251)	(0.283)
Constant	-16.708***	-19.454***	-17.667***	-16.865***	-18.922***	-19.407***	-20.604***	-17.131***
	(0.556)	(0.736)	(0.870)	(0.656)	(0.295)	(0.653)	(0.394)	(0.441)
Observations	9,247	8,633	5,365	3,882	9,247	8,633	5,365	3,882
R-squared	0.701	0.710	0.723	0.671	0.700	0.710	0.724	0.674

The dependent variable is the first difference of turnout. Robust standard errors in parentheses ;*** p<0.01, ** p<0.05, * p<0.1

Table S6: **Determinants of ΔTurnout as a function of distance to clusters**

VARIABLES	(1) All	(2) All	(3) Young	(4) Old	(5) All	(6) All	(7) Young	(8) Old
Distance from Covid-19 cluster (km)	0.001	0.007***	-0.002***	0.003***				
	(0.000)	(0.001)	(0.001)	(0.001)				
Within 20 km of a Cluster					-0.212	-0.624**	0.131	-1.362*
					(0.268)	(0.264)	(0.290)	(0.703)
Δ Population over 75 (%)	-0.402***	-0.285***	-0.482***	-0.445***	-0.401***	-0.273***	-0.476***	-0.444***
	(0.064)	(0.067)	(0.092)	(0.092)	(0.064)	(0.067)	(0.092)	(0.093)
Δ Population 25 or younger (%)	-0.200***	-0.226***	-0.177***	-0.297***	-0.197***	-0.224***	-0.183***	-0.269***
	(0.052)	(0.054)	(0.064)	(0.087)	(0.052)	(0.054)	(0.064)	(0.087)
Δ Population 65_74 (%)	-0.012	0.046	-0.107*	0.098	-0.008	0.047	-0.128**	0.120
	(0.048)	(0.051)	(0.063)	(0.074)	(0.048)	(0.051)	(0.062)	(0.074)
Δ Registered Voters (log)	-8.178***	-7.335***	-7.388***	-8.471***	-8.186***	-7.438***	-7.486***	-8.665***
	(0.561)	(0.565)	(0.736)	(0.746)	(0.561)	(0.570)	(0.747)	(0.744)
Δ Median Income	0.140*	0.557***	-0.057	0.385***	0.144*	0.566***	-0.088	0.399***
	(0.077)	(0.088)	(0.095)	(0.139)	(0.077)	(0.088)	(0.096)	(0.139)
Change in number of lists running (unchanged as reference):								
From 1 to 2	15.953***	15.943***	16.456***	15.166***	15.948***	15.923***	16.463***	15.149***
	(0.213)	(0.223)	(0.265)	(0.354)	(0.213)	(0.223)	(0.265)	(0.352)
From 1 to 3 and more	17.037***	17.193***	17.625***	16.411***	17.039***	17.161***	17.590***	16.380***
	(0.504)	(0.540)	(0.670)	(0.760)	(0.503)	(0.540)	(0.674)	(0.746)
From 2 to 1	-14.994***	-14.824***	-15.191***	-14.656***	-14.993***	-14.826***	-15.196***	-14.659***
	(0.160)	(0.164)	(0.207)	(0.252)	(0.161)	(0.164)	(0.206)	(0.255)
From 2 to 3 and more	3.283***	3.197***	3.504***	2.982***	3.291***	3.194***	3.471***	3.016***
	(0.207)	(0.209)	(0.288)	(0.300)	(0.207)	(0.209)	(0.288)	(0.299)
From 3 and more to 1	-15.847***	-15.763***	-16.647***	-14.816***	-15.842***	-15.757***	-16.686***	-14.830***
	(0.335)	(0.347)	(0.460)	(0.462)	(0.335)	(0.348)	(0.459)	(0.467)
From 3 and more to 2	1.069***	1.087***	0.955***	1.113***	1.077***	1.089***	0.922***	1.122***
	(0.189)	(0.192)	(0.251)	(0.285)	(0.189)	(0.193)	(0.250)	(0.286)
Constant	-19.426***	-22.092***	-18.943***	-20.011***	-19.338***	-21.516***	-19.187***	-19.510***
	(0.151)	(0.348)	(0.188)	(0.265)	(0.142)	(0.347)	(0.181)	(0.237)
Region FE	No	Yes	No	No	No	Yes	No	No
Observations	9,247	8,633	5,365	3,882	9,247	8,633	5,365	3,882
R-squared	0.700	0.710	0.724	0.671	0.700	0.709	0.723	0.670

The dependent variable is the first difference of turnout. Robust standard errors in parentheses ; *** $p<0.01$, ** $p<0.05$, * $p<0.1$

Table S7: **Determinants of 2020 Turnout under COVID-19 Pandemic**

VARIABLES	(1)	(2)	(3)	(4)	(5)	(6)
Distance from COVID-19 cluster (km)	0.012***	0.012***	0.013***	0.013***	0.012***	0.007***
	(0.001)	(0.001)	(0.001)	(0.001)	(0.001)	(0.003)
Population over 75 (%)	0.244***	0.176***	0.098***	0.117***	0.265***	0.158***
	(0.029)	(0.028)	(0.029)	(0.028)	(0.029)	(0.028)
Population 25 or younger (%)	0.002	-0.023	0.026	0.035	0.040	-0.047
	(0.035)	(0.035)	(0.034)	(0.033)	(0.036)	(0.035)
Population 65_74 (%)	-0.054	-0.012	-0.068*	-0.017	-0.089**	-0.089**
	(0.040)	(0.039)	(0.039)	(0.038)	(0.041)	(0.038)
Number of lists running (3 and more as reference):						
An unique list	-19.051***	-19.112***	-19.163***	-19.080***	-18.979***	-18.738***
	(0.203)	(0.200)	(0.198)	(0.195)	(0.203)	(0.191)
Two lists	-0.454**	-0.721***	-0.815***	-0.518***	-0.424**	-0.276
	(0.186)	(0.182)	(0.181)	(0.177)	(0.185)	(0.174)
Incumbent mayor running (1 if yes)	0.368***	0.363***	0.412***	0.429***	0.354***	0.234*
	(0.135)	(0.134)	(0.133)	(0.131)	(0.135)	(0.124)
Macron Vote	0.183***	0.195***	0.202***	0.197***	0.178***	0.022
	(0.016)	(0.016)	(0.016)	(0.016)	(0.016)	(0.020)
Pop. density (log)	-5.355***	-6.693***	-6.776***	-4.892***	-8.768***	-5.624***
	(0.175)	(0.121)	(0.114)	(0.165)	(0.804)	(0.182)
Homeowners pop (%)	0.045***	0.020**	0.048***	0.037***	0.030***	0.018**
	(0.008)	(0.008)	(0.008)	(0.008)	(0.009)	(0.008)
Educated pop (%)	0.178***	0.138***	0.204***	0.188***	0.169***	-0.034
	(0.031)	(0.031)	(0.031)	(0.030)	(0.031)	(0.033)
Median Income	-0.366***	-0.300***	-0.277***	-0.208***	-0.373***	-0.177***
	(0.024)	(0.025)	(0.024)	(0.024)	(0.025)	(0.030)
Unemployment rate	0.011	0.099**	0.093**	0.166***	-0.007	-0.266***
	(0.040)	(0.040)	(0.040)	(0.039)	(0.040)	(0.056)
Hotel Rooms/capita (log)	17.443***	16.629***	13.047***	14.236***	16.283***	16.167***
	(3.107)	(3.029)	(2.826)	(2.748)	(3.011)	(2.841)
Registered Voters (log)					3.522***	
					(0.818)	
City size according to inhab., 4 evenly groups, first one (least populated) as reference):						
Second group	-1.390***			-1.249***	-1.462***	-1.384***
	(0.190)			(0.186)	(0.192)	(0.179)
Third group	-3.283***			-2.838***	-3.459***	-3.200***
	(0.228)			(0.238)	(0.233)	(0.223)
Forth group	-5.113***			-4.488***	-5.408***	-4.859***
	(0.372)			(0.369)	(0.381)	(0.370)
City type (Rural city as reference):						
Center of agglomeration		0.269		0.165		
		(0.190)		(0.188)		
Suburb of agglomeration		1.890***		1.900***		
		(0.193)		(0.200)		
Isolated city		3.005***		1.867***		
		(0.170)		(0.177)		
City category (City of large agglomeration as reference):						
Belonging to multiple large agglomeration			1.036***	0.703***		
			(0.230)	(0.231)		
Belonging to med. and small urban area			1.757***	2.149***		
			(0.228)	(0.231)		
Belonging to multiple other agglomeration			3.969***	3.097***		
			(0.242)	(0.246)		
Outside any agglomeration			5.083***	4.072***		
			(0.304)	(0.307)		
Constant	92.210***	100.088***	97.443***	81.706***	93.160***	101.845***
	(2.271)	(2.047)	(2.020)	(2.183)	(2.283)	(2.391)
Department FE	No	No	No	No	No	Yes
Observations	9,247	9,247	9,247	9,247	9,247	9,247
R-squared	0.731	0.735	0.738	0.748	0.732	0.776

Robust standard errors in parentheses; *** p<0.01, ** p<0.05, * p<0.1

Table S8: **Determinants of Turnout using various measures related to COVID-19**

VARIABLES	(1)	(2)	(3)	(4)	(5)	(6)
Distance from COVID-19 cluster (km)	0.013***					
	(0.001)					
Virus incidence in depart. (1 if yes)		-18.282***				
		(0.108)				
Total Emergency Visits			-0.000			
			(0.000)			
COVID-19 Emergency Visits				-0.263***		
				(0.005)		
COVID-19 Hospitalizations					0.003	
					(0.010)	
COVID-19 Medical Acts						-0.025***
						(0.006)
Population over 75 (%)	0.206***	0.184***	0.213***	0.227***	0.213***	0.167***
	(0.032)	(0.020)	(0.032)	(0.030)	(0.032)	(0.045)
Population 25 or younger (%)	-0.934***	-0.219***	-1.090***	-0.869***	-1.092***	-1.146***
	(0.037)	(0.023)	(0.036)	(0.034)	(0.036)	(0.047)
Population from 65 to 74 (%)	-2.171***	-0.153***	-2.217***	-1.902***	-2.216***	-2.233***
	(0.043)	(0.030)	(0.043)	(0.041)	(0.043)	(0.056)
Number of lists running (3 and more as reference):						
An unique list	-15.640***	-15.713***	-15.755***	-15.975***	-15.752***	-16.130***
	(0.225)	(0.142)	(0.227)	(0.212)	(0.227)	(0.290)
Two lists	-0.666***	-0.188	-0.737***	-0.740***	-0.736***	-0.984***
	(0.199)	(0.126)	(0.201)	(0.188)	(0.201)	(0.252)
Incumbent mayor running (1 if yes)	-10.041***	-1.002***	-10.020***	-8.024***	-10.022***	-10.316***
	(0.191)	(0.132)	(0.193)	(0.185)	(0.193)	(0.250)
Macron Vote	0.305***	0.186***	0.336***	0.269***	0.339***	0.282***
	(0.018)	(0.011)	(0.018)	(0.017)	(0.018)	(0.022)
Pop. density (log)	-5.100***	-4.806***	-5.189***	-4.723***	-5.203***	-5.068***
	(0.193)	(0.122)	(0.196)	(0.183)	(0.196)	(0.241)
Homeowners pop (%)	0.158***	0.084***	0.156***	0.148***	0.156***	0.121***
	(0.008)	(0.005)	(0.009)	(0.008)	(0.009)	(0.011)
Educated pop (%)	0.049	0.359***	0.209***	0.244***	0.209***	0.181***
	(0.034)	(0.021)	(0.034)	(0.032)	(0.034)	(0.043)
Median Income	-0.702***	-0.435***	-0.783***	-0.604***	-0.789***	-0.530***
	(0.025)	(0.016)	(0.026)	(0.023)	(0.026)	(0.031)
Unemployment	0.411***	0.324***	0.629***	0.599***	0.623***	0.741***
	(0.044)	(0.027)	(0.044)	(0.040)	(0.043)	(0.052)
Hotel Rooms per capita (log)	24.434***	13.579***	22.664***	21.528***	22.691***	18.187***
	(2.608)	(1.652)	(2.636)	(2.463)	(2.637)	(3.007)
City size according to inhab., 4 evenly groups, first one (least populated) as reference:						
Second group	-0.421**	-0.704***	-0.427**	-0.427**	-0.427**	-0.504*
	(0.204)	(0.129)	(0.206)	(0.193)	(0.206)	(0.274)
Third group	-1.150***	-2.025***	-1.156***	-1.275***	-1.155***	-1.304***
	(0.254)	(0.161)	(0.257)	(0.240)	(0.257)	(0.331)
Forth group	-2.120***	-3.185***	-1.994***	-2.229***	-1.994***	-2.404***
	(0.418)	(0.265)	(0.423)	(0.395)	(0.423)	(0.525)
Constant	142.184***	107.397***	146.710***	132.821***	146.898***	146.156***
	(2.452)	(1.564)	(2.486)	(2.323)	(2.470)	(3.126)
Observations	18,608	18,608	18,608	18,608	18,608	11,350
R-squared	0.575	0.830	0.565	0.621	0.565	0.554

Robust Standard errors in parentheses; *** p<0.01, ** p<0.05, * p<0.1

Table S9: **Determinants of Turnout, Autoregressive Model**

VARIABLES	(1) turnout	(2) turnout	(3) turnout	(4) turnout	(5) turnout	(6) turnout
Lagged turnout	0.356***	0.342***	0.339***	0.334***	0.355***	0.297***
	(0.008)	(0.008)	(0.008)	(0.008)	(0.008)	(0.008)
Distance from COVID-19 cluster (km)	0.008***	0.008***	0.008***	0.008***	0.008***	0.004*
	(0.001)	(0.001)	(0.001)	(0.000)	(0.001)	(0.003)
Population over 75 (%)	0.141***	0.089***	0.025	0.051**	0.145***	0.085***
	(0.026)	(0.026)	(0.026)	(0.026)	(0.026)	(0.026)
Population 25 or younger (%)	0.011	-0.015	0.023	0.035	0.019	-0.049
	(0.031)	(0.031)	(0.031)	(0.030)	(0.032)	(0.032)
Population from 65 to 74 (%)	-0.055	-0.028	-0.072**	-0.031	-0.062*	-0.087**
	(0.035)	(0.035)	(0.035)	(0.035)	(0.036)	(0.035)
Number of lists running (3 and more as reference):						
An unique list	-17.880***	-17.992***	-18.052***	-17.983***	-17.867***	-17.832***
	(0.183)	(0.183)	(0.182)	(0.179)	(0.184)	(0.179)
Two lists	-0.182	-0.463***	-0.560***	-0.249	-0.176	-0.084
	(0.163)	(0.162)	(0.161)	(0.158)	(0.163)	(0.158)
Incumbent mayor running (1 if yes)	0.118	0.122	0.158	0.183	0.116	0.056
	(0.121)	(0.121)	(0.120)	(0.118)	(0.121)	(0.115)
Macron Vote	0.116***	0.129***	0.135***	0.128***	0.116***	0.013
	(0.014)	(0.014)	(0.015)	(0.014)	(0.015)	(0.018)
Pop. density (log)	-4.111***	-5.635***	-5.762***	-3.849***	-4.823***	-4.614***
	(0.153)	(0.111)	(0.105)	(0.147)	(0.713)	(0.165)
Homeowners pop. (%)	-0.005	-0.022***	-0.001	-0.007	-0.008	-0.014*
	(0.007)	(0.007)	(0.007)	(0.007)	(0.008)	(0.008)
Educated pop (%)	0.086***	0.056**	0.109***	0.101***	0.084***	-0.052*
	(0.028)	(0.028)	(0.028)	(0.027)	(0.028)	(0.030)
Median Income	-0.215***	-0.179***	-0.161***	-0.107***	-0.217***	-0.135***
	(0.022)	(0.022)	(0.022)	(0.021)	(0.022)	(0.028)
Unemployment	-0.112***	-0.048	-0.047	0.010	-0.115***	-0.252***
	(0.036)	(0.037)	(0.036)	(0.036)	(0.036)	(0.052)
Hotel Rooms per capita (log)	13.076***	12.363***	9.605***	10.712***	12.844***	12.718***
	(2.688)	(2.717)	(2.691)	(2.609)	(2.696)	(2.503)
Registered Voters (log)					0.732	
					(0.724)	
City size according to inhab., 4 evenly groups, first one (least populated) as reference:						
Second group	-1.489***			-1.362***	-1.504***	-1.394***
	(0.171)			(0.168)	(0.172)	(0.166)
Third group	-3.390***			-2.973***	-3.426***	-3.172***
	(0.201)			(0.214)	(0.206)	(0.204)
Forth group	-5.291***			-4.733***	-5.352***	-4.897***
	(0.324)			(0.329)	(0.331)	(0.335)
City type (Rural city as reference):						
Center of agglomeration		0.011		-0.026		
		(0.171)		(0.169)		
Suburb of agglomeration		0.957***		1.134***		
		(0.174)		(0.180)		
Isolated city		2.318***		1.303***		
		(0.154)		(0.162)		
City category (City of large agglomeration as reference):						
Belonging to multiple large agglomeration			0.517**	0.316		
			(0.201)	(0.202)		
Belonging to med. and small urban area			1.244***	1.679***		
			(0.206)	(0.209)		
Belonging to multiple other agglomeration			3.167***	2.509***		
			(0.219)	(0.223)		
Outside any agglomeration			4.005***	3.236***		
			(0.273)	(0.276)		
Constant	64.028***	74.844***	73.421***	58.056***	64.282***	78.010***
	(2.056)	(1.897)	(1.881)	(2.014)	(2.071)	(2.220)
Department FE	No	No	No	No	No	Yes
Observations	9,247	9,247	9,247	9,247	9,247	9,247
R-squared	0.785	0.784	0.786	0.794	0.785	0.809

Robust standard errors in parentheses ; p<0.01, ** p<0.05, * p<0.1

Table S10: **Turnout and age by distances from COVID-19 clusters**

Sample Within:	< 50 km<br>(1)	50-100 km<br>(2)	100-200 km<br>(3)	200-600 km<br>(4)	< 50 km<br>(5)	50-100 km<br>(6)	100-200 km<br>(7)	200-600 km<br>(8)
Post	-18.580***	-21.533***	-23.143***	-23.280***	-17.621***	-19.429***	-21.385***	-21.026***
	(1.075)	(1.046)	(0.890)	(0.874)	(0.657)	(0.637)	(0.542)	(0.549)
Population over 65 (%)	0.371***	0.292***	0.100***	0.179***	0.333***	0.236***	0.032*	0.079***
	(0.042)	(0.033)	(0.026)	(0.022)	(0.027)	(0.024)	(0.019)	(0.016)
Post × Population over 65 (%)	-0.119*	0.029	0.112***	0.118***	-0.165***	0.006	0.096***	0.111***
	(0.062)	(0.052)	(0.043)	(0.038)	(0.038)	(0.033)	(0.027)	(0.023)
Median Income					-0.166***	-0.144***	-0.116***	-0.088**
					(0.022)	(0.029)	(0.028)	(0.034)
Unemployment					-0.728***	-0.095*	-0.054	0.123***
					(0.064)	(0.057)	(0.039)	(0.039)
Number of lists running (3 and more as reference):								
Unique Liste					-15.724***	-15.473***	-15.742***	-15.316***
					(0.321)	(0.319)	(0.244)	(0.239)
Two Lists					-0.024	0.406	-0.025	-0.502**
					(0.293)	(0.292)	(0.220)	(0.203)
Registered Voters (log)					-6.407***	-6.784***	-7.065***	-6.721***
					(0.140)	(0.163)	(0.116)	(0.111)
Constant	57.281***	59.990***	64.526***	66.438***	123.091***	121.143***	127.471***	124.416***
	(0.702)	(0.617)	(0.503)	(0.476)	(1.705)	(1.827)	(1.350)	(1.299)
Observations	3,146	3,802	5,944	5,602	3,146	3,802	5,944	5,602
R-squared	0.531	0.533	0.534	0.547	0.830	0.829	0.835	0.824

Robust standard errors in parentheses; *** p<0.01, ** p<0.05, * p<0.1

Table S11: **The effect of distance on turnout, Conley standard errors**

	Dependent variable: Turnout		
	(1)	(2)	(3)
	All	Old	Young
Post	-19.987***	-21.265***	-19.875***
	(0.244)	(0.470)	(0.293)
	[0.524]	[0.530]	[0.537]
Distance from COVID-19 cluster (km)	0.022***	0.015***	0.023***
	(0.001)	(0.001)	(0.001)
	[0.003]	[0.003]	[0.003]
Post × Distance from COVID-19 cluster (km)	-0.001	0.004**	-0.005***
	(0.001)	(0.002)	(0.002)
	[0.001]	[0.002]	[0.002]
Constant	62.661***	65.461***	62.009***
	(0.172)	(0.372)	(0.195)
	[0.902]	[0.927]	[0.910]
Observations	18,496	7,074	11,294
R^2	0.544	0.534	0.560
F Statistic	7,367.055*** (df = 3; 18492)	2,697.317*** (df = 3; 7070)	4,798.081*** (df = 3; 11290)
	(df = 3; 18492)	(df = 3; 7070)	(df = 3; 11290)

Robust standard errors are in parentheses, Conley standard errors are in brackets; *** p<0.01, ** p<0.05, * p<0.1

Table S12: **Variables definitions and sources (I)**

**Variables**	**Definition**	**Level**	**Source**
	**COVID-19 measures**		
Cluster	Dummy variable coding for whether the municipality is a cluster. A municipality is identified as a cluster of COVID-19, if it has been identified/labelled as such by the national press as of March 15th.	municipality	Various
Distance to Cluster	Distance to the nearest COVID-19 cluster in kilometers	municipality	Various
Number COVID Cases	Cumulative number of coronavirus cases per department as of the 14th of March 2020.	Department	ARS
Number COVID Deaths	Cumulative number of coronavirus deaths per department as of the 14th of March 2020.	Department	ARS
Total Emergency Visits	Total amount of emergency room visits on the 14th of March 2020.	Department	SPF
COVID-19 Emergency Visits	Number of emergency room visits for suspicion of COVID-19 on the 14th of March 2020.	Department	SPF
COVID-19 Hospitalizations	Number of hospitalizations among emergency department visits for suspicion of COVID-19 on the 14th of March 2020.	Department	SPF
Total Medical Acts	Total number of medical acts (SOS Medecins) on the 14th of March 2020.	Department	SPF
COVID-19 Medical Acts	Number of medical acts (SOS Medecins) on the 14th of March 2020.	Department	SPF
Virus Dummy	Dummy variables indicating whether there is at least one coronavirus case in the department as of the 14th of March 2020.	Department	SPF
	**Municipal elections and political variables**		
Registered Voters	Number of registered voters for the municipal elections of 2014 and 2020.	municipality	Min
Ballots	Number of actual voters for the municipal elections of 2014 and 2020.	municipality	Min
Turnout	Number of actual voters divided by the number of registered voters, computed for the municipal elections of 2014 and 2020.	municipality	Min
Number of Lists	Number of lists running for the municipal election of 2014 or 2020.	municipality	Min
Incumbent Candidate	Dummy variable coding for whether one of the lists is ran by the current major of the municipality. To create this variable, we match the names from the elected majors files with the names of candidates to the first round of the municipal elections.	municipality	Min
Macron Vote	Percentage of votes cast for Macron at the first round of the 2017 presidential election.	municipality	Min

ARS stands for *Agences Régionales de Santé, SPF for Santé Publique France*, and Min for *Ministère de l'Intérieur*.

Table S13: **Variables definitions and sources (II)**

**Variables**	**Definition**	**Level**	**Source**
	**Sociodemographic and Geographic Indicators**		
Pop 65 or More	Percentage of the population of 65 or more, based on the 2011 (2016) census for the municipal election of 2014 (2020). For the 2008 municipal election, we use the census data of 2008.	municipality	INSEE
Pop 75 or More	Percentage of the population of 75 or more, based on the 2011 (2016) census for the municipal election of 2014 (2020).	municipality	INSEE
Pop 25 or Less	Percentage of the population of 25 or less, based on the 2011 (2016) census for the municipal election of 2014 (2020).	municipality	INSEE
Pop 65 to 74	Percentage of the population between 65 and 74, based on the 2011 (2016) census for the municipal election of 2014 (2020).	municipality	INSEE
Population	Number of inhabitants based on the 2011 (2016) census for the municipal election of 2014 (2020).	municipality	INSEE
City size	We create four groups of city, each containing 25% of the city, according to their place within the population's distribution	municipality	INSEE
Population Density	Density of the population based on the 2011 (2016) census for the municipal election of 2014 (2020).	municipality	INSEE
Median Income	Median income based on the 2014 (2017) survey for the municipal election of 2014 (2020).	municipality	INSEE
Unemployment Rate	Unemployment rate as defined by International Labour Office, in the employment area as of 2013 (2019) for the municipal election of 2014 (2020).	municipality	INSEE
% Homeowner	Percentage of the population that owns its home, based on the 2011 (2016) census for the municipal election of 2014 (2020).	municipality	INSEE
% Educated	Percentage of the population with at least a high school diploma, based on the 2011 (2016) census for the municipal election of 2014 (2020).	municipality	INSEE
Number Hotel Rooms	Number of hotel rooms in the commune as of 2020. Data collected from the tourism equipment dataset.	municipality	INSEE
Municipality Status	Variable indicating whether the commune is (1) a rural area, (2) a city-center, (3) a suburb, or (4) a remote city. Information comes from the 2017 dataset on urban units	municipality	INSEE
Municipality Category	Variable indicating whether the municipality is (1) a remote city or a monomunicipality urban unit, (2) an intradepartmental agglomeration (3) an interdepartmental agglomeration (4) an interregional agglomeration, (5) an international agglomeration. Information comes from the 2017 dataset on urban units.	municipality	INSEE
Number Homes Elderly	Amount of homes for elderly dependent persons as of 2014 (2018) for the municipal election of 2014 (2020).	municipality	INSEE

INSEE stands for *Institut National de la Statistique et des Etudes Economiques*

References

[1] Riker WH, Ordeshook PC. A Theory of the Calculus of Voting. American political science review. 1968;62(01):25–42.

[2] Blais A. To Vote or Not to Vote ? The Merits and Limits of Rational Choice Theory. Pittsburgh: University of Pittsburgh Press; 2000.

[3] Feddersen T. Rational choice theory and the paradox of not voting. Journal of Economic Perspective. 2004;38:390–413.

[4] Geys B. Explaining voter turnout: A review of aggregate-level research. Electoral Studies. 2006;25(4):637–663.

[5] Geys B. Rational theories of voter turnout: a review. Political Studies Review. 2006;4(1):16–35.

[6] Adman P. Does poor health cause political passivity even in a Scandinavian welfare state? Investigating the impact of self-rated health using Swedish panel data. Electoral Studies. 2020;65:102110. Available from: http://www.sciencedirect.com/science/article/pii/S0261379418305237.

[7] Bukov A, Maas I, Lampert T. Social participation in very old age: Cross-sectional and longitudinal findings from BASE. The Journals of Gerontology Series B: Psychological Sciences and Social Sciences. 2002;57(6):P510–P517.

[8] Denny KJ, Doyle OM. ?? Take up thy bed, and vote? Measuring the relationship between voting behaviour and indicators of health. The European Journal of Public Health. 2007;17(4):400–401.

[9] Kelleher C, Timoney A, Friel S, McKeown D. Indicators of deprivation, voting patterns, and health status at area level in the Republic of Ireland. Journal of Epidemiology & Community Health. 2002;56(1):36–44.

[10] Mattila M, Söderlund P, Wass H, Rapeli L. Healthy voting: The effect of self-reported health on turnout in 30 countries. Electoral Studies. 2013;32(4):886–891.

[11] Reitan TC. Too sick to vote? Public health and voter turnout in Russia during the 1990s. Communist and Post-Communist Studies. 2003;36(1):49–68.

[12] Söderlund P, Rapeli L. In sickness and in health: Personal health and political participation in the Nordic countries. Politics and the Life Sciences. 2015;34(1):28–43.

[13] Bhatti Y, Hansen KM. Retiring from voting: Turnout among senior voters. Journal of Elections, Public Opinion & Parties. 2012;22(4):479–500.

[14] Ojeda C, Pacheco J. Health and voting in young adulthood. British Journal of Political Science. 2019;49(3):1163–1186.

[15] Schur LA, Kruse DL. What determines voter turnout?: Lessons from citizens with disabilities. Social Science Quarterly. 2000;p. 571–587.

[16] Schur L, Shields T, Kruse D, Schriner K. Enabling democracy: disability and voter turnout. Political Research Quarterly. 2002;55(1):167–190.

[17] Schur L, Adya M. Sidelined or Mainstreamed? Political Participation and Attitudes of People with Disabilities in the U nited S tates. Social Science Quarterly. 2013;94(3):811–839.

[18] Schur L, Ameri M, Adya M. Disability, voter turnout, and polling place accessibility. Social Science Quarterly. 2017;98(5):1374–1390.

[19] Ojeda C. Depression and political participation. Social Science Quarterly. 2015;96(5):1226–1243.

[20] Sund R, Lahtinen H, Wass H, Mattila M, Martikainen P. How voter turnout varies between different chronic conditions? A population-based register study. J Epidemiol Community Health. 2017;71(5):475–479.

[21] Gollust SE, Rahn WM. The bodies politic: chronic health conditions and voter turnout in the 2008 election. Journal of health politics, policy and law. 2015;40(6):1115–1155.

[22] Mattila M, Wass H, Lahtinen H, Martikainen P. Sick leave from work and the voting booth? A register-based study on health and turnout. Acta Politica. 2018;53(3):429–447.

[23] Leininger A, Schaub M. Voting at the dawn of a global pandemic. 2020;.

[24] Merkley E, Bridgman A, Loewen PJ, Owen T, Ruths D, Zhilin O. A Rare Moment of Cross-Partisan Consensus: Elite and Public Response to the COVID-19 Pandemic in Canada. Canadian Journal of Political Science/Revue canadienne de science politique. 2020;p. 1–12.

[25] Amat F, Arenas A, Falcó-Gimeno A, Muñoz J. Pandemics meet democracy. Experimental evidence from the COVID-19 crisis in Spain. 2020;.

[26] BOL D, GIANI M, BLAIS A, LOEWEN PJ. The effect of COVID-19 lockdowns on political support: Some good news for democracy? European Journal of Political Research;forthcoming.

[27] Urbatsch R. Influenza and Voter Turnout. Scandinavian Political Studies. 2017;40(1):107–119.

[28] Noury AG. Abstention in daylight: Strategic calculus of voting in the European Parliament. Public Choice. 2004;121(1-2):179–211.

[29] Matsusaka JG. Election closeness and voter turnout: Evidence from California ballot propositions. Public Choice. 1993;76(4):313–334.

[30] Rosenthal H, Sen S. Electoral participation in the French fifth republic. American Political Science Review. 1973;67(1):29–54.

[31] Papke LE, Wooldridge JM. Econometric methods for fractional response variables with an application to 401 (k) plan participation rates. Journal of applied econometrics. 1996;11(6):619–632.
